# Evaluation of Phosphopolyoxometalates with Mixed Addenda (Mo, W, V) as Corrosion Inhibitors for Steels

**DOI:** 10.3390/ma16247600

**Published:** 2023-12-11

**Authors:** Gabriela Elena Badea, Alexandrina Fodor, Anda Ioana Grațiela Petrehele, Ioana Maior, Monica Toderaș, Claudia Mona Morgovan

**Affiliations:** 1 Department of Chemistry, Faculty of Informatics and Sciences, University of Oradea, 1 Universitatii Str., 410087 Oradea, Romania; gbadea@uoradea.ro (G.E.B.); afodor@uoradea.ro (A.F.); andapetrehele@yahoo.com (A.I.G.P.); cmorgovan@yahoo.com (C.M.M.); 2 Department of Inorganic Chemistry, Physical Chemistry and Electrochemistry, Faculty of Chemical Engineering and Biotechnologies, National University of Science and Technology POLITEHNICA Bucharest, 313 Splaiul Independentei, 060042 Bucharest, Romania; 3 Department of Physics, Faculty of Informatics and Sciences, University of Oradea, 1 Universitatii Str., 410087 Oradea, Romania

**Keywords:** S235 steel, SS304 steel, corrosion inhibitor, passivation, polyoxometalates

## Abstract

Nowadays, choosing a corrosion inhibitor is not only based on efficiency, but must also consider the toxicity of the compound, the impact on the environment, and, obviously, the regulations in the field. In the last two decades, a special class of substances has begun to be studied, namely polyoxometalates (POMs). Their electronic properties and redox characteristics make the polyoxometalates potential candidates to be used in many electrochemical processes, and as potential corrosion inhibitors. Electrochemical methods such as a Tafel extrapolation plot, chronopotentiometry, or gravimetry have been used to establish the capacity of corrosion inhibition of S235 and SS304 steels in the presence of phosphovanadomolibdate acid (@PMoV) and phosphovanadotungstate acid (@PWV) in 0.5 M sulphuric acid solution. The inhibition efficiency for S235 steel is about 90.6% for @PMoV, and 69.5% for @PWV after 24 h of immersion. In the case of SS304 steel, polyoxometalates have similar effects: the inhibition degree, as a function of Flade potential, is 4.66 for @PMoV; better than 3.26 for @PWV, with both proving the passivant effect.

## 1. Introduction

**Figure 1:**
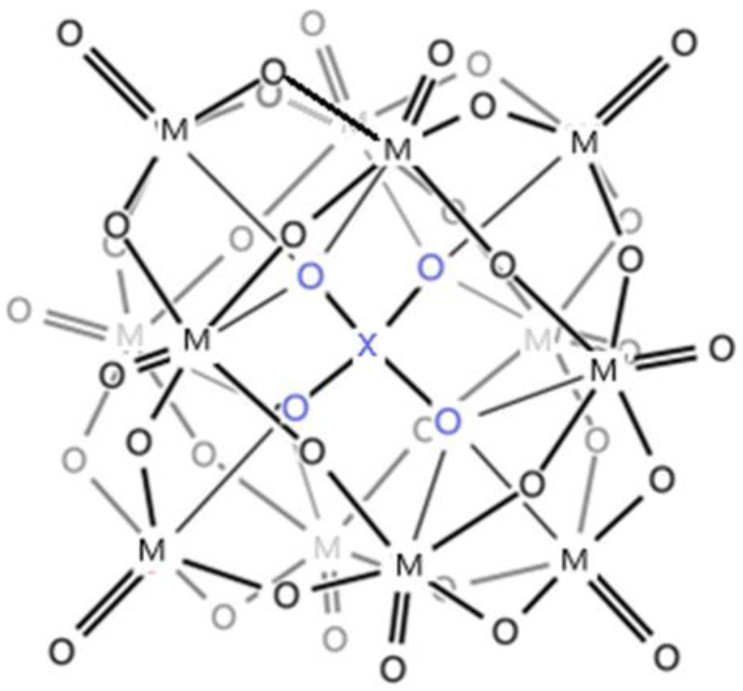
Structure of Keggin polyoxometalates, [XM_12_O_40_]^n−^.

POMs are a class of inorganic compounds, and they are important for theoretical and practical interest [1,2,3,4,5,6,7,8,9,10,11,12,13,14,15,16,17,18,19,20,21,22,23,24,25,26,27,28,29,30,31,32,33,34,35,36,37,38,39,40,41]. The polyoxometalates can be described as cluster anions obtained by condensation reactions under acidic conditions of transition-metal oxoanions (principally those in groups V b and VI b in a high oxidation state—especially Mo, V and W) and metalloid anions in a high oxidation state (especially P, Si, and As). POMs determine a tremendous amount of scientific research and applications [1,2] with great emphasis on those with a Keggin structure [XM_12_O_40_]^n−^ (where the metal M could be W or Mo, and X could be tetrahedral anions as PO_4_^3−^), composed of elementary metal oxides {MOx} (the metal M is called the addenda atom and x could take values between 4 and 7) [3,4,5]. These structures are stable only in an acid medium [6]. In Figure 1 is presented the structure of [XM_12_O_40_]^n−^, Keggin polyoxometalates [30,31,36,39,40]. Many POMs, with different magnetic centers, determined a lot of structures with different magnetic behaviors [8,9,10]. For example, a Cr-based POM showed deviations from typical paramagnetic behavior [9,10]. Some polyoxometalates used in medicine, for anticancer drugs, are based on magnetic nanoparticles [11]. Macroporous polyoxometalate hybrids present catalytic activity, stability, and recyclability for the oxidative desulfurization [12,13].

POMs based on tungsten (VI, V) or molybdenum (VI, V) also presented catalytic activity in some redox electrochemical processes [14,31] or in electrochemical energy storage [15,16]. In organic chemistry, POMs present catalytic activity in a large spectrum of chemical, electrochemical or photochemical reactions [17].

The M=O bond exhibits significant π-electron donation capabilities, which therefore result in polyoxometalates (POMs) displaying remarkable redox characteristics [18]. The transition from the oxidized to the reduced state is reversible [6,18]. Numerous prospective uses of immobilized polyoxometalates (POMs) on solid substrates have been explored in the field of detection sensors, namely for the identification and quantification of analytes in gas or liquid phases. These applications are primarily built upon the technique of depositing POMs onto solid substrates, employing methods such as chemisorption and electrochemical deposition [7]. These properties that can suggest that POMs can act as an important player in the electrochemistry field: corrosion inhibitors [32,34], electrocatalyst [33], electroactive materials [35], electrochemical sensors [38], cathode material [41], or as a catalyst in different organic reactions [17,37].

Conducting polymers doped with polyoxometalates were reported as hybrid material which might be an efficient barrier against corrosion [20,21,22]. Polypyrrole/phosphotungstate coating presents good corrosion resistance on mild steel [23] and on low alloy steel in seawater [24]. Polypyrrole films doped with tungstate, deposited on mild steel [25], provide a good protection against corrosion. Another class of materials, like POMs, are the metal–organic frameworks, tested to be an important thin-film coating and protection against corrosion [26].

Polyoxometalates are inorganic compounds that contain oxygen and transition metals. They have catalytic, optical, and electrochemical properties that make them useful for various applications. One of these applications is the inhibition of steel corrosion in acidic environments, such as those used in the oil or chemical industry. Corrosion is a process of deterioration of metals caused by a reaction with the environment. It reduces the strength and durability of steel and can lead to economic and environmental losses. Polyoxometalates can prevent or slow down corrosion by forming a protective layer on the metal surface, which prevents contact with corrosive agents. In addition, polyoxometalates can modify the electrochemical properties of the metal, reducing the corrosion potential and the reaction rate.

The general importance of the use of polyoxometalates as corrosion inhibitors of steels in acidic environments is evident from numerous studies and some previous research carried out in this field.

The specific importance of using polyoxometalates with a higher content of molybdenum ions is given by the fact that they have greater stability in acidic environments and a greater affinity for the metal surface. This leads to a greater efficiency of corrosion inhibition and a reduction in the amount of polyoxometalates needed to obtain the desired effect.

The use of an organic–inorganic hybrid with molybdenum proved to present an inhibitory effect for mild steel protection, when working in HCl media [27]. Similar compounds, the polyoxometallates H_5_[PMo_10_V_2_O_40_] and H_5_[PW_10_V_2_O_40_], present two reversible redox couples in a former cycling voltammetry study, which proves that they can have a potentially oxidizing action on metals [6,29].

In the present work, electrochemical investigations were performed to establish the behavior of two steels: S235—a mild steel, and SS304—a stainless steel, in sulphuric acid in the presence of some phosphopolyoxometalates with mixed addenda (Mo, W, V): H_6_[PMo_9_V_3_O_40_] or @PMoV and H_6_[PW_9_V_3_O_40_] or @PWV, conducted to reveal the corrosion inhibitor action of these POMs. A previous study indicated that some other phosphopolyoxometalates from the same family, H_5_[PMo_10_V_2_O_40_] and H_5_[PW_10_V_2_O_40_], act as oxidizing anions [29]. This research incorporates various electrochemical approaches, namely potentiostatic polarization, chronopotentiometry, and weight loss analysis.

## 2. Materials and Methods

### 2.1. Materials

The inhibition properties of polyoxometalates acids @PMoV and @PWV were studied on S235 and SS304 steels, with the following composition, in weight percentage, wt% (Table 1):

The exposed area of the metal specimens was 1 cm^2^. The steel samples underwent surface treatment by a mechanical cleaning method, specifically by sanding with abrasive paper, of varying grit sizes, followed by degreasing with carbon tetrachloride.

Prior to conducting the experiments, the electrodes went through a degreasing process to ensure a clean surface. The S235 steel electrode was immersed in a 0.5 M H_2_SO_4_ solution for a duration of 2 min, while the SS304 electrode was treated for 5 min at 60 °C in an activation solution, made of 15% HNO_3_ + 2% HF (volume%).

All experiments were conducted in 0.5M H_2_SO_4_ in the presence of @PMoV and @PWV as corrosion inhibitors in different molar concentrations (10^−2^M; 10^−3^M; 10^−4^M, and 10^−5^M).

Solutions of sulfuric acid were produced using annular grade reagent and double-distilled water. The polyoxometalates were synthesized in the laboratory. All experimental procedures were conducted under controlled conditions at a temperature of 20 ± 1 °C.

The Mo, W, V, and P elemental analysis was carried out with the assistance of a Varrian ASA 220 spectrophotometer. Thermal analyses carried out in an air environment on a Paulik-Erdely OD-103 derivatograph, with a heating rate of 5 °C per minute in the temperature range of 20–800 °C, was used to quantify the number of water molecules. Using KBr pellets and a Bio-Rad FTS 60A spectrophotometer (Bio-Rad Digilab Division, Texas City, TX, USA), Fourier Transform Infrared (FT-IR) spectra were collected in the wavenumber range of 400–4000 cm^−1^.

The graphic representations and part of the calculations were made with Microsoft Excel and Origin Lab programs (Microsoft Excel 365 and Origin 2023B programs).

### 2.2. Preparation of Heteropolyoxometalates

#### 2.2.1. Synthesis of @PMoV (H_6_[PMo_9_V_3_O_40_]∙34H_2_O)

A quantity of 5.82 g (0.03 mol) NaVO_3_∙4H_2_O was dissolved in 50 mL boiling distilled water in a Berzelius beaker. A solution prepared by dissolving 1.6 g (0.0115 mol) NaH_2_PO_4_∙H_2_O in 10 mL distilled water is added over the metavanadate solution. The resulting solution is brought to room temperature, and under stirring, 96% H_2_SO_4_ is added in drops to pH 2.0–2.5. The resulting red solution is mixed with a solution prepared by dissolving 21.78 g (0.09 mol) Na_2_MoO_4_∙2H_2_O in 30 mL distilled water. Both 50% and 96% H_2_SO_4_ solution were utilized in order to bring about the necessary pH adjustments in the reaction mixture, which brought the value to approximately pH = 2.0. After allowing the heated solution to cool down for half an hour at room temperature, the free POM acid was extracted from the solution using a separating funnel and four fractions, each of which contained 25 mL of diethyl ether. POM etherate was freed of ether at room temperature and the red residue was passed in a minimum distilled water in an evaporating dish into a vacuum desiccator over concentrated sulphuric acid for two days. A quantity of 14.65 g of H_6_[PMo_9_V_3_O_40_]∙34H_2_O was obtained (yield 63.5% based on V) and the molecular mass M = 2305.8 g∙mol^−1^. (Anal calc. for PMoV: P, 1.34%; Mo, 37.45%; V, 6.63%; Found: P, 1.3%; Mo, 37.5%; V, 6.6%; FT-IR bands found in KBr disk: 3350 s, 1642 s, 1055 ms, 958 s, 863 s, 780, 765 cm^−1^). TG data: weight loss of 26.5% corresponding to 33.94 H_2_O molecules.

#### 2.2.2. Synthesis of @PWV (H_6_[PW_9_V_3_O_40_]∙14H_2_O)

The synthesis of the trilacunary, α-Na_8_[HPW_9_O_34_]∙11H_2_O, was carried out as following: In a Berzelius beaker, 5.82 g (0.03 mol) of NaVO_3_∙4H_2_O was dissolved in 80 mL of distilled water under continuous stirring just at the boiling point. In the solution cooled at room temperature, 96% H_2_SO_4_ was added under continuous stirring until pH 3.0 was reached. To the reaction mixture, 26.12 g (0.01 mol) α-Na_8_[HPW_9_O_34_]∙11H_2_O was gradually added while the pH was maintained from 2.5–3 by adding 96% H_2_SO_4_ at the same time. Finally, the orange-red solution of @PVW was brought to pH 2–2.5 and allowed to react at 60 °C for one hour. The @PVW acid formed is separated as an etherate and passed back from the aqueous solution to the protonated form, as in the case with @PMoV acid synthesis. A quantity of 19.73 g H_6_[PW_9_V_3_O_40_]∙14H_2_O was obtained and the molecular mass M = 2736.6 (yield 72.1% based on V). (Anal calc. for PWV: P, 1.13%; W, 60.46%; V, 5.58%; Found: P, 1.15%; W, 60.5%; V, 5.5%; FT-IR bands found in KBr disk: 3480 s, 1628 s, 1059 ms, 962 s, 870 s, 795, 765 cm^−1^). TG data: weight loss of 9% corresponding to 13.68 H_2_O molecules.

### 2.3. Potentiostatic Polarization Method

Corrosion behaviors of S235 steels were investigated by the following methods: potentiostatic polarization (both anodic and cathodic curves for the mild steel S235, and only anodic for SS304, with a rate of 40 mV/3 min), chronopotentiometry, and weight loss.

The 173 PAR-potentiostat was utilized in order to carry out electrochemical experiments. Experiments were conducted using a three-electrode cell that was partitioned into two separate compartments. All potentials were determined relative to a saturated calomel electrode (SCE), which was separated from the working electrode in a separate compartment via a Luggin capillary.

Calculating the corrosion rate of the metal specimens based on the potentiostatic polarization curves required taking into account the corrosion current density, $i_{corr}^{0}$. This was accomplished by linear extrapolation of anodic and cathodic Tafel slopes of the polarization curves. The inhibition efficiency, IE%, was calculated with the formula:

(1) $$IE(\%)=\frac{i_{corr}^{0}-i_{corr}}{i_{corr}^{0}}\;\cdot \;100$$

where $i_{corr}^{0}$ is the corrosion current density without inhibitor and $i_{corr}$ with inhibitor.

### 2.4. Chronopotentiometry Method

The graphical representations demonstrate how the open circuit corrosion potential, or E_corr_, changed over the course of time based on the results of measurements taken on the two different samples of steel, S235 and SS304, in 0.5 M H_2_SO_4_ without and with the studied polyoxometalates, @PMoV and @PWV, in various molar concentrations, in 500 min.

### 2.5. Weight Loss Method

The metal samples were withdrawn from the solutions after 24 h of immersion, then washed, dried, and weighed. Using the differences in weight, the weight loss, Δ*m* (g), and the corrosion rate, *K* (g/m^2^h), were calculated using the following equations:

(2) $$∆m=m_{i}-m_{f}$$

(3) $$K=\frac{∆m}{S\times t}$$

where *m_i_* is the initial weight of metal specimen, *m_f_* is the weight after 24 h of immersion, *S* is the surface area exposed to corrosion, and *t* is the immersion time, in hours. In the case of mild steels, a time of 24 h is enough to allow the formation of the inhibitor protective film, which would sufficiently reduce the corrosion rate. Every experiment was performed three times and the presented data in tables are the average values.

## 3. Results and Discussions

In general, the substances used as catalysts or inhibitors, including corrosion inhibitors, are substances that modify the kinetics of chemical reactions, acting on their mechanism. Corrosion inhibitors and/or passivants, in particular, are substances that must be added in very small quantities to stop or control the phenomenon of corrosion. Using a very small amount is a necessary requirement not only from the point of view of costs, but also from the point of view of impurity and/or chemical reactivity. The minimum concentration of the inhibitor, at which the corrosion rate of the steel does not change, represents the solution in the analyzed case study. This is the reason why the concentration of solutions goes from 10^−2^ M to a very low one, 10^−6^ M.

### 3.1. S235 Steel Behavior in the Presence of @PMoV and @PWV

Between the corrosion rate of a metal, *v_cor_*_,_ and the corrosion current density, *i_cor_*, there is a direct proportional relationship; the constant being *k_e_*—the electrochemical equivalent of the corroding material (iron in the present case, being the base metal for steels):

(4) $$v_{cor}=k_{e}\cdot i_{cor}$$

Electrochemical processes, including metal corrosion (metal oxidation (anodic branch) and the reduction reaction of the depolarizer (cathode branch)), are described by the Butler–Volmer equation (Equation (5)). It expresses the exponential dependence of the current density on the electrode potential:

(5) $$i_{cor}=i_{0}\cdot e^{-\frac{\alpha zF}{RT}\cdot (E-E_{cor})}$$

in which: *i*_0_ is the exchange current densities for steel; *α*—the charge transfer coefficients for the partial anodic and cathodic reactions; *z*—the number of electrons transferred in the corrosion process; *R*—general gas constant; *T*—absolute temperature, and the difference (*E* − *E_cor_*) is the charge transfer polarization corresponding to metal corrosion.

Basically, the Butler–Volmer equation can be written simplified, in the logarithmic form:

(6) $$\mathrm{lg}i_{cor}=\mathrm{lg}i_{0}-k\;\cdot (E-E_{cor})$$

**Figure 2:**
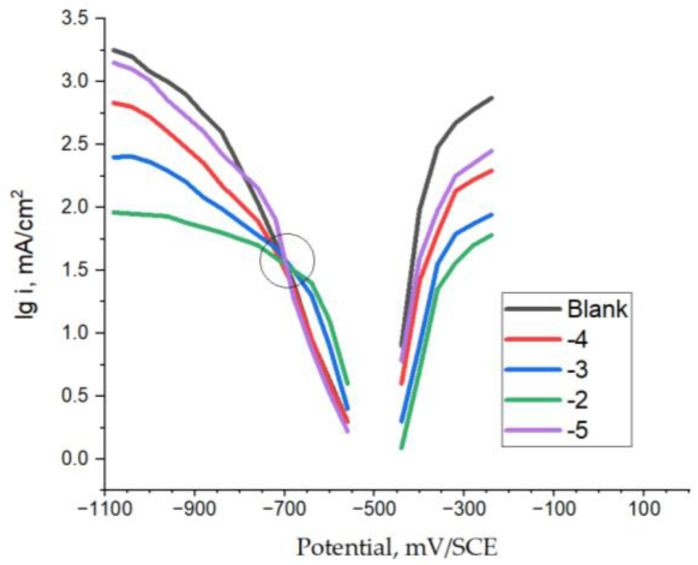
Polarization anodic and cathodic curves for S235-0.5M H_2_SO_4_-@PMoV, at various lg C, M.

**Figure 3:**
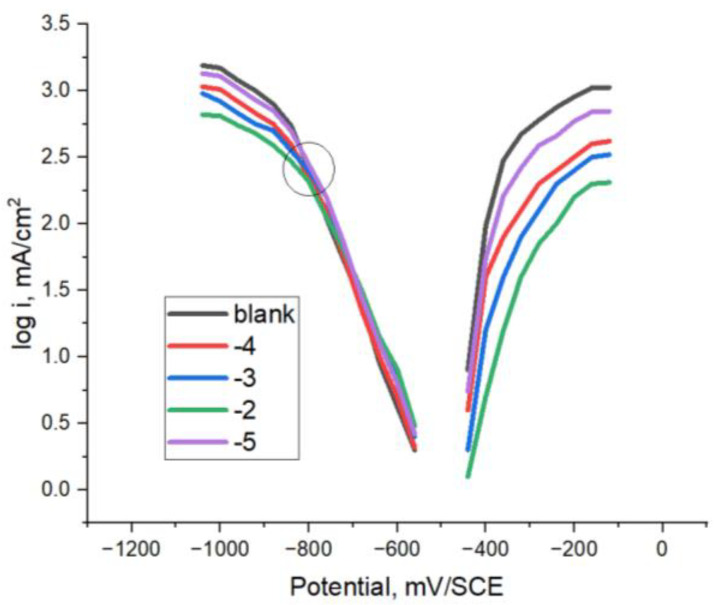
Polarization anodic and cathodic curves for S235-0.5M H_2_SO_4_-@PWV, at various lg C, M.

Both anodic and cathodic curves were recorded when S235 steel was polarized in potentiostatic conditions in 0.5 M sulfuric acid, as well as those obtained in the acid solutions with additions of polyoxometalates @PMoV and @PWV, at various molar concentration, M. The results obtained for the two additives are graphically represented in Figure 2 and Figure 3.

**Figure 4:**
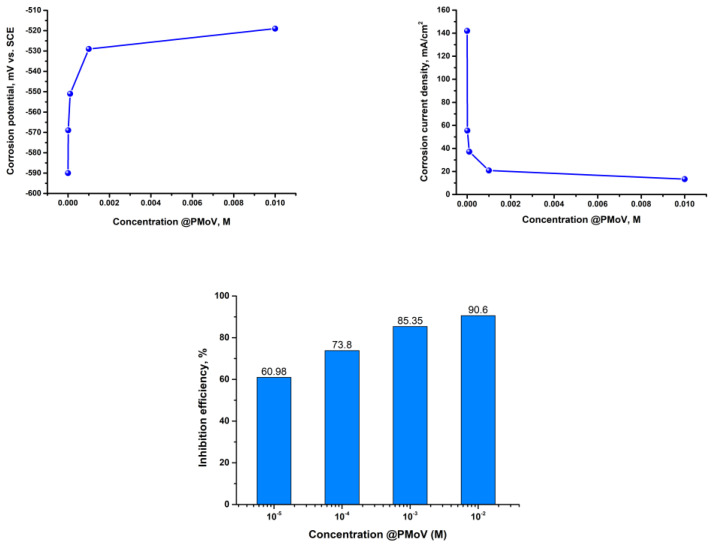
Polarization results for S235-@PMoV in 0.5 M H_2_SO_4._

**Figure 5:**
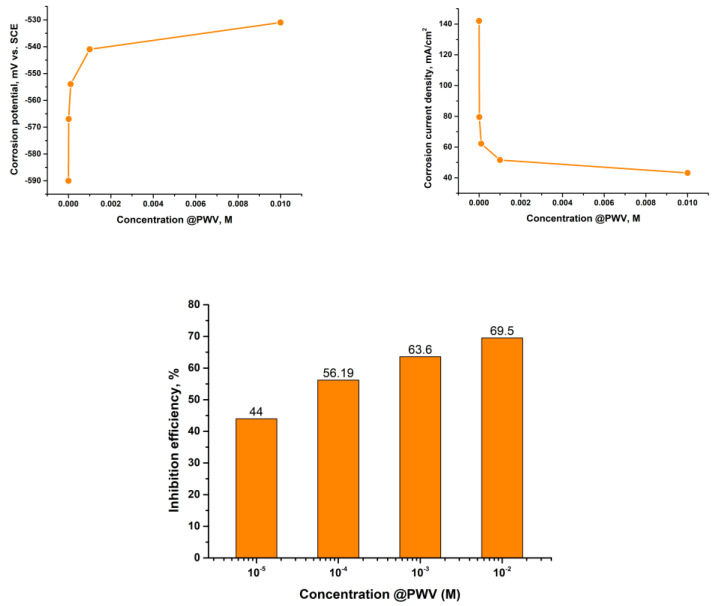
Polarization results for S235-@PWV in 0.5 M H_2_SO_4._

The electrochemical characteristics determined from the extrapolation of linear Tafel slopes exhibited in the polarization curves are presented in Figure 4 and Figure 5 for @PMoV and for @PWV, respectively. The metrics encompassed within this study consist of the corrosion potential, *E_corr_*, the density of the corrosion current, *i_corr_*_,_ and the inhibition efficiency, IE, expressed as a percentage. In all instances investigated, it was seen that a decrease in the rate of corrosion was accompanied by an augmentation in the effectiveness of inhibition.

In both Figure 2 and Figure 3, the appearance of an isopotential point can be noted on the cathodic branch, more pronounced in the case of @PMoV. The equipotential points, where several curves meet, indicate a phenomenon related to the degree of coverage of the electrode correlated to the irreversible blocking of the electrode surface, independent of the potential, which would be explained by the stronger adsorption of the oxidant anion of @PMoV, compared to that of @PWV. This behavior leads to the reduction in the cathodic current density and, implicitly, of the corrosion current density. Similar results have been obtained for other related polyoxometalates [29].

The value of the measured density of corrosion current in the absence of polyoxometalates (142 mA/cm^2^) is lower than in the case of carbon steel (CS, 148 mA/cm^2^) [29], mainly due to the composition of the steel; the former having copper in its composition, so, in principle, being less active than the second. As expected, and according to the results from the experimental data, the density of corrosion current in the sulphuric acid solution is very high for the S235 electrode, and it is close to the one obtained for carbon steel in a previous study [29]. Despite experiencing a slight reduction in size, due to the presence of the investigated polyoxometalates, it retains significant magnitudes.

**Figure 6:**
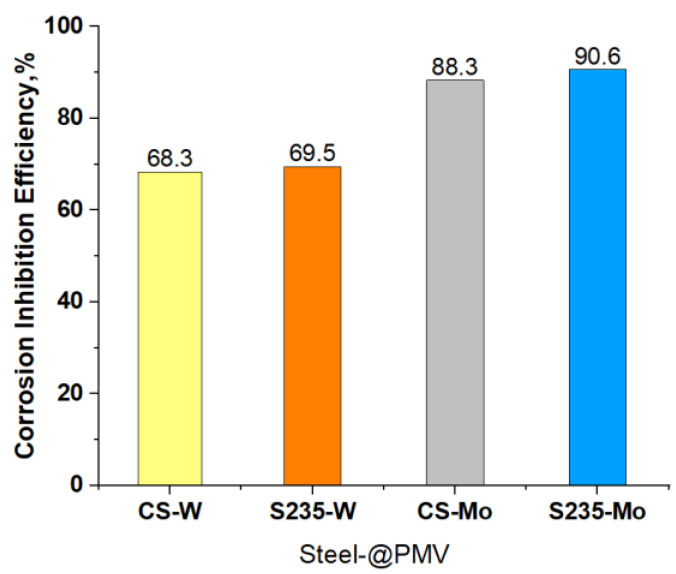
Comparative corrosion inhibition efficiency of Mo and W based polyoxometalates on CS and S235 mild steels.

The level of Inhibition efficiency demonstrates an upward trend as the concentration of the inhibitor increases. However, it remains inadequate, even when the concentration of polyoxometalates reaches 0.01 M. The corrosion potentials exhibit a gradual trend towards more positive values, particularly in the instance of @PMoV, which demonstrates inhibition efficiencies that are 21.1% greater than those of @PWV. The comparative inhibition efficiencies, IE%, of the studied polyoxometalates for different mild steels [29] are presented in Figure 6.

These results are also sustained by another class of tungsten (W)-based polyoxometalates, which have been reported to have inhibitor efficiencies of 81–96.9% for carbon steel [34].

The general corrosion process for a metal in acid media could be described by two partial reactions, taking place at the same potential, with the same rate:

- Anodic reaction: M-ze → M^z+^
- Cathodic reaction: H^+^ + 1e → 1/2H_2._

In acid media, the cathodic reaction is the reduction of the H^+^ ion formed by acid molecule dissociation. The two partial conjugated electrochemical processes that are components of the corrosion process take place independently on the metal surface. However, they are related by the shared potential at which they take place, which is denoted by the symbol *E_corr_*, and the same rate of development, which is denoted by the symbol *i_corr_*.

The polyoxometalates that were investigated perform the role of cathodic inhibitors, as suggested by the electrochemical mechanism. These inhibitors work by inhibiting the cathodic partial reaction, which in turn slows down the overall process of corrosion. Polyoxometallates @PMoV and @PWV, which operate as cathodic inhibitors, increase the reduction overvoltage of hydrogen ions, and so function as an oxidizing agent.

**Figure 7:**
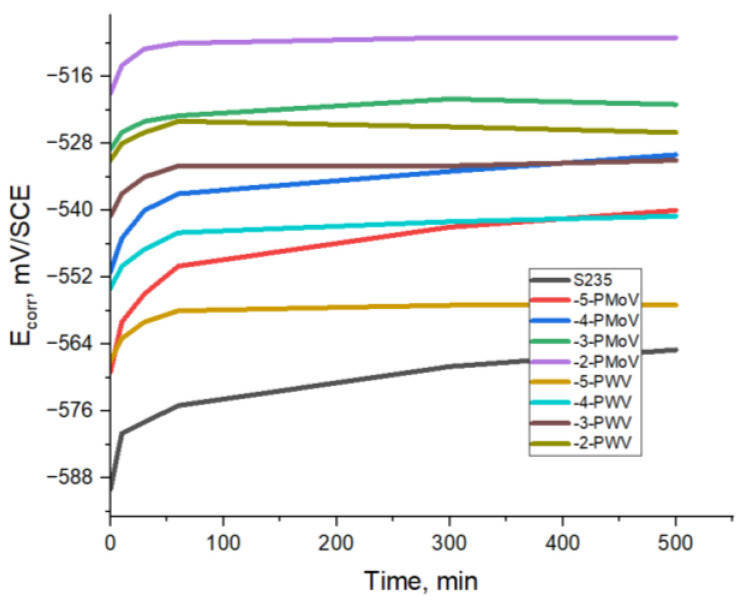
Chronopotentiometric curves for S235-0.5M H_2_SO_4_ with @PMoV and @PWV at various lg C, M.

Figure 7 depicts the outcomes of chronopotentiometry studies, referring to the fluctuation of corrosion potentials over a duration of 500 min, in an open circuit condition, for the S235—0.5 M H_2_SO_4_ system. The observed variations are dependent upon the molar concentrations of @PMoV and @PWV, denoted as lg C, M.

In both scenarios, the potentials exhibit a gradual shift towards more electropositive values. This trend persists until reaching a steady state after a duration of about 100 min. The observed shift of the corrosion potential in the positive direction indicates that the compounds under investigation have the potential to act as corrosion inhibitors. Nevertheless, it is important to acknowledge that a relatively large quantity of these substances is required in comparison to traditional inhibitors. However, it is evident that the S235 steel remains within the active domain, indicating that the inclusion of the additive inhibitors, specifically @PMoV and @PWV, in an acidic environment and at the amounts examined, hardly assures the conditions for the self-passivation of S235. Based on the findings from the previous investigations, namely polarization and chronopotentiometry, it can be deduced that the corrosion inhibitors suggested for S235 steel primarily function as cathodic inhibitors. The phenomenon can be explained by the adsorption of polyoxometalates on the surface of S235 samples and the formation of adsorption films or even oxidation products that act as a protective barrier, leading to important changes in the anodic and, especially, cathodic current density values.

**Figure 8:**
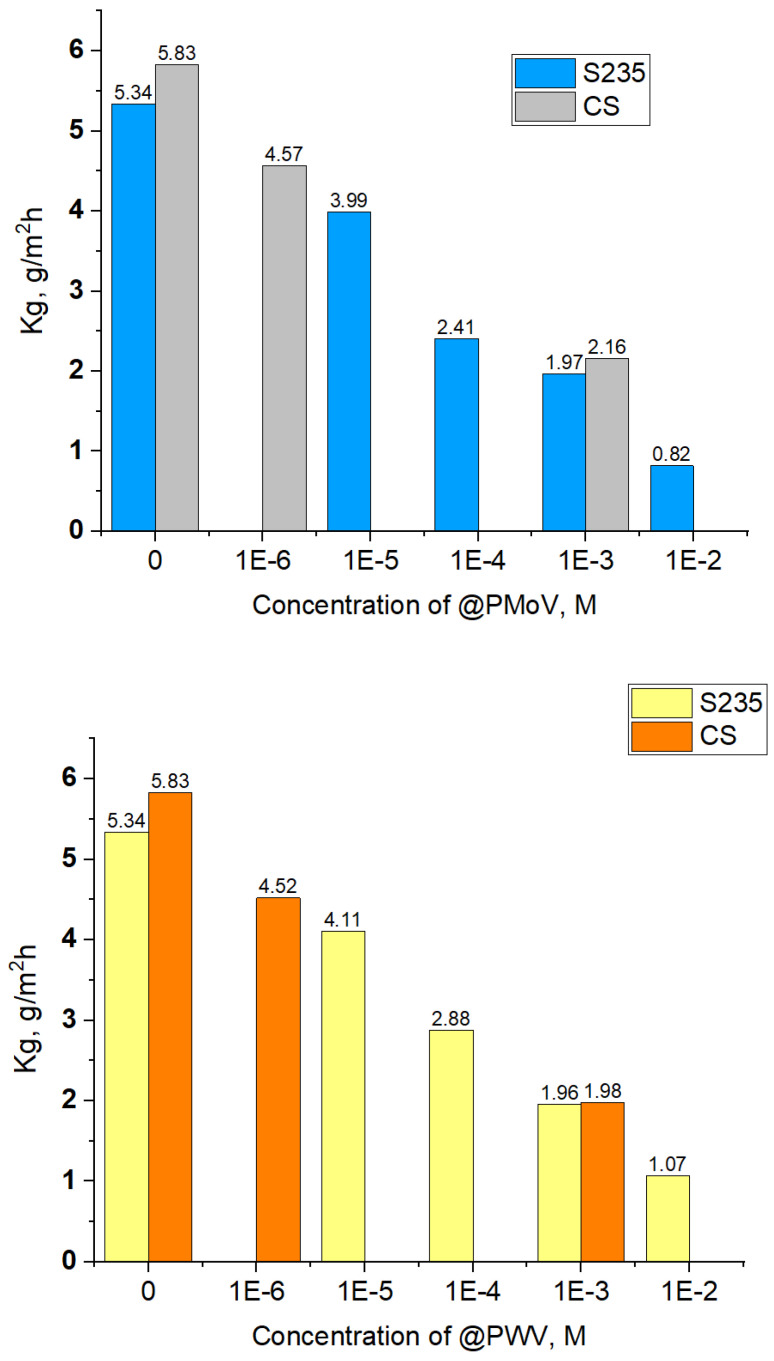
Comparative corrosion rates of CS [29] and S235 mild steels in 0.5 M H_2_SO_4_ with Mo and W based polyoxometalates with various concentrations C, M.

The characteristics of S235 steel in the studied corrosion media are further supported through the findings obtained from the weight loss analysis conducted on the S235 steel specimens following 24 h of immersion. Figure 8 presents comparative corrosion rates obtained on mild steels, S235 and CS [29], when using Mo and W based polyoxometalates in various molar concentrations.

The corrosion rates exhibit a gradual decline as the concentration of PMoV increases, suggesting that PMoV may serve as a more effective inhibitor compared to PWV. However, it is worth noting that even at a level of 0.01 M, the corrosion rates remain considerable.

Previous research on CS yielded results that were similar but not identical to these ones [29]. However, while the results obtained by the gravimetric method support the slight inhibitory effect of the new polyoxometalates, they are not entirely conclusive because the corrosion rate of S235 compared to CS is lower, even for sulfuric acid solution without additives. The studies must be developed, using other inhibitors from the same family of polyoxometalates on the same metal support, to establish which exactly is most responsible for the better behavior in acidic media corrosion.

### 3.2. SS304 Steel Behavior in the Presence of @PMoV and @PWV

**Figure 9:**
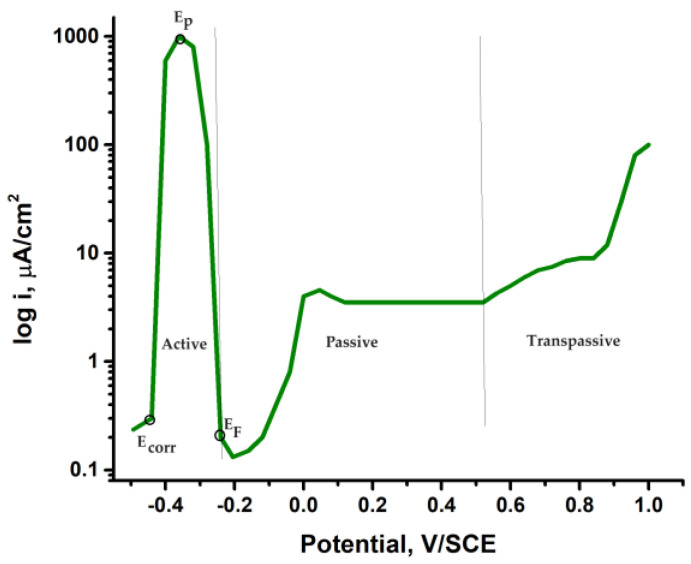
Passivation curve for stainless steel 18/10 in 0.5 M H_2_SO_4_ [29].

The passivity of metals refers to the condition of a metal when it is subjected to an environment where corrosion is thermodynamically possible, but the actual corrosion process is hindered due to kinetic factors. In this present state, the metallic substrates exhibit a high degree of resistance to corrosion and demonstrate electrochemical behavior comparable to that of noble metals, rendering them immune to attack. The phenomenon under consideration, which is evident in metallic substrates such as iron, chromium, nickel, and their respective alloys, including stainless steels, can be attributed to the formation of a thinner oxide layer on the metal’s surface. This film serves as a protective barrier, effectively separating the metal from its surrounding environment, hence enhancing its durability. The object achieves a state of being impervious, exhibiting no discernible alterations on its exterior. The process of achieving this state in a metal is accomplished through anodic or spontaneous passivation. Anodic passivation is achieved by applying an external electric current, which results in the movement of the potential towards increasingly positive values. The development of the formed film is attributed to electrochemical reactions occurring at the interface between the metal and solution. The extent of solubility of the initial anodic product plays a crucial role in this process. In the context of stainless steels, it is observed that the first anodic product exhibits a high solubility, hence necessitating the attainment of high current densities in order to achieve the passive state. The curves obtained on both SS304 and SS steels are almost identical, indicating that they are very similar steels. The provided information is illustrated in Figure 9, which shows the conventional anodic polarization curve featuring the active–passive transition of a stainless steel 10Ni/18Cr [29].

When the potential is shifted in the positive direction, the dissolution rate in the active–passive region reaches a maximum value at a specific potential known as the critical passivation potential. Beyond this point, the dissolution rate becomes largely unaffected by further increases in potential, indicating the attainment of a passive state. Subsequently, in the transpassive region, the current begins to increase once again. The Flade potential is commonly referred to as the reactivation potential in the context of reverse polarization. The consolidation of the passive film occurs within the range bounded by the passivation potential and the Flade potential.

The electrochemical parameters of the corrosion of stainless steel (SS) measured in a 0.5 M H_2_SO_4_ solution and calculated from a potentiostatic polarization curve, are given in Table 2.

**Figure 10:**
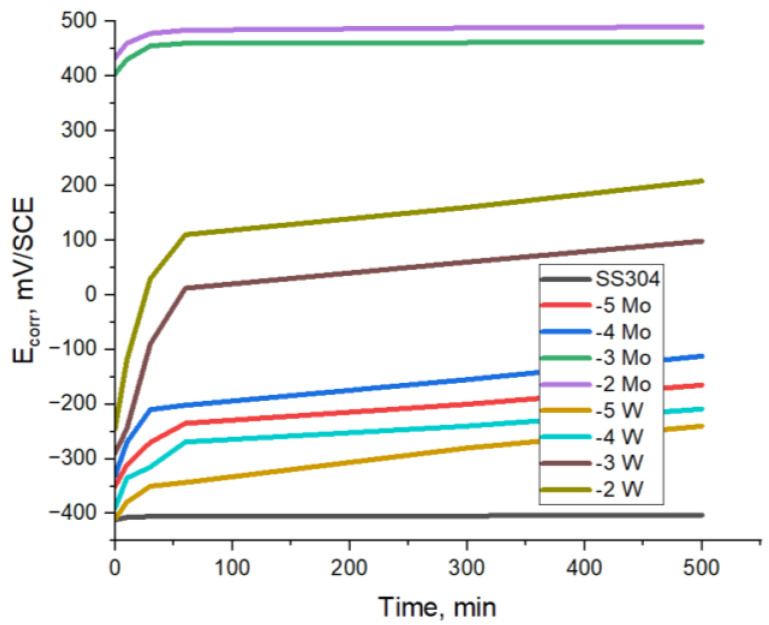
Chronopotentiometric curves for SS304–0.5M H_2_SO_4_ with @PMoV and @PWV in various molar concentration, lg C.

The cathodic current values observed on the passive surfaces do not exhibit significant variations in response to the presence or absence of dissolved oxygen. This can be attributed to the fact that the transport of dissolved oxygen through the 0.5 M H_2_SO_4_ solution occurs at the limiting diffusion current, which is considerably lower than the critical passivation current. In this particular scenario, the existence of an oxidizing ion, such as polyoxometalate ions, plays a role in establishing the passive state. The chronopotentiometric assays conducted on SS304 stainless steel were applied to examine its behavior in both acidic environments and solutions containing @PMoV and @PWV, as shown in Figure 10.

Stainless steel corrosion potentials’ dependence on immersion time, in 0.5 M H_2_SO_4_, in the absence and in the presence of different concentrations of heteropolyoxometalates acids, are presented in Figure 10.

High-alloy steels including chromium and chromium–nickel, such as ferritic steel with 18% chromium; austenitic steel with 8% nickel and 18% chromium; or austenitic steel with 18% chromium, 18% manganese, and 2% nickel; exhibit passivation behavior similar to that of iron, chromium, and nickel. These steels generate an oxide film that possesses electronic conductivity. Nonetheless, the polarization behavior of the material is significantly impacted by the characteristics and qualities of the alloying elements. For instance, the value of the Flade potential varies according to the chromium content. The explanation for the ideal qualities of these steels extends beyond the structural variations, namely austenitic or ferritic, and also includes the composition of the steels and the presence of passivating coatings.

Indeed, this study is not comprehensive in nature, and further analyses and investigations are required. The significance of the current work lies in the discovery of a specific class of compounds, or alternatively, a single compound, that offers the most effective option for protecting the examined stainless steel from corrosion.

When the process of metal passivation occurs, the metal surface is covered with a protective oxide layer. When an optimal thickness is reached, the corrosion rate no longer increases, as in the case of active dissolution, but tends asymptotically to a constant value. A bigger value of the slope would indicate an active corrosion, and no inhibition effect. The obtained curves, in Figure 7 and Figure 10, initially grow rapidly, form a shoulder, and then stabilize at an almost constant value. This stabilization of the corrosion potential indicates the effectiveness of both the inhibitor and passivant inhibitor. The fact that the potential remains practically constant after 500 min of immersion in the aggressive environment is the most important argument which attests to the passivation role of polyoxometalate ions.

The experiment involved observing the behavior of SS304 steel, which had its surface activated through a 5 min treatment at 60 °C using an activation solution consisting of 15% HNO_3_ and 2% HF. It was found that even after being immersed for 500 min, the SS304 steel remained within the active zone, with a potential close to the corrosion potential of approximately −411 mV/SCE. This potential was slightly more positive than the corrosion potential of SS steel, which was measured to be −430 mV/SCE [29]. The experiments were conducted with the addition of two polyoxometalates, specifically @PMoV and @PWV. It was observed that the anodic potential shifted towards more electropositive values, with a more pronounced shift observed in the presence of the compound containing Molybdenum. This shift was followed by the establishment of a steady state, indicating the attainment of passivity, within the range of −250 mV/SCE to +490 mV/SCE for @PMoV, to +208 mV/SCE for @PWV. The findings align with the observed trend in the prior investigation [29], specifically demonstrating an enhanced efficacy that is distinctly evident in solutions with inhibitor concentrations of 10^−3^ and 10^−2^ M.

The fact that both polyoxometalates exhibited variations in corrosion potentials within the passive range provides data that indicate they both work as passivant inhibitors within the particular settings that were investigated. After an immersion period of 500 min, noticeable modifications were not found on the surfaces of the stainless steel specimens, nor were there any variations in the color of the solution. In addition, there were no obvious shifts in the chemical composition of the solution. The polioxometalate compound @PMoV was proved to be better than @PWV also in this case.

A measure of the ability of an additive to show passivant properties can be quantitatively calculated using the “inhibition degree”, *Z*, described by Equation (4):

(7) $$Z=\frac{\pi_{0}-\pi_{i}}{\pi_{0}}$$

in which: *π*_0_ and *π_i_* is the product *i_corr_* × (*E_F_* − *E_corr_*) in the absence (*o*) and the presence of the inhibitor (*i*).

**Figure 11:**
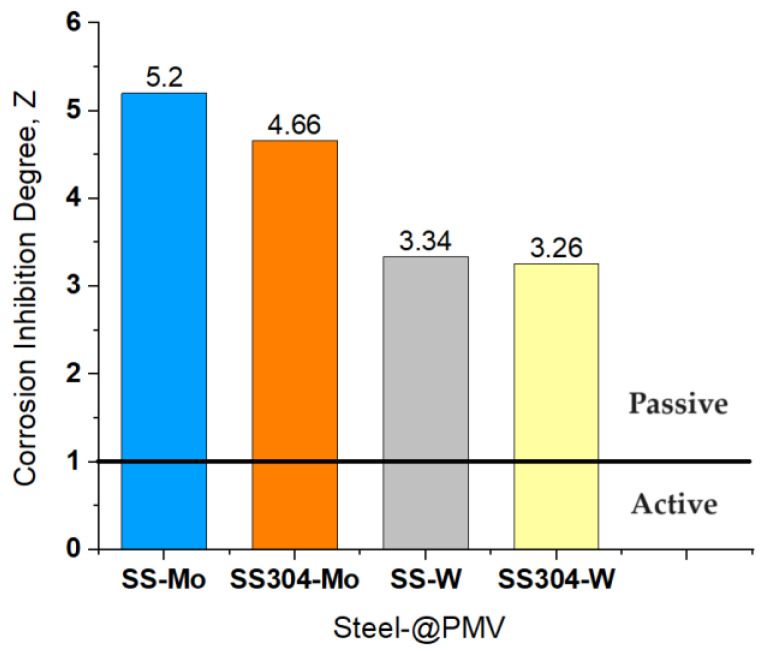
Comparative inhibition degree, Z, for SS and SS304 steels in H_2_SO_4_ + 0.01M Mo and W based polyoxometalates.

The self-passivation of a metal surface, generated by the presence of an oxidizing chemical species, is described by values of the degree of inhibition, *Z* ≥ 1. Figure 11 presents comparatively the values obtained for inhibition degree, for a solution with 0.01 M polyoxometalate; this last concentration being proved to be the best among the studied solutions.

The acquired data reinforce that, even in the context of mild steels, polyoxometalate containing Mo exhibits a more effective protective effect. This finding demonstrates that Mo serves as an oxidizing species with passivating capabilities that surpass those of W.

## 4. Conclusions

The originality of this study consists in highlighting the protective effect against corrosion of some Keggin-type polyoxometalates based on Mo and W for mild steel S235 and austenitic stainless steel SS304 through specific mechanisms: cathodic corrosion inhibitor in the case of S235, and both passivator and promoter of passivity film for SS304 steel. The results are in accordance with a previous study [29], carried out on similar metals and similar compounds, which opens up new perspectives that need to be investigated: finding the best polyoxometalate ion as inhibitor for a specific metal, in a given environment. The study highlights that the working algorithm for the investigation of electrochemical parameters is a correct and viable one, but it will require in the future to be completed with additional information related to the composition and structure of the metals and their passive films, but also to the stability of the polyoxometalate in different conditions.

It is well known that Mo and W–POMs could influence the supported metal centers in ways different from the simple dispersion on the electrode area. This could lead to an affectation of the chemisorptive properties. The studied polyoxometalates could induce, at low potentials, modifications in the generation of –OH groups, causing oxidation of passivating CO adsorbates [24]. Despite numerous studies, the subject is far from being exhausted. The ability of polyoxometalates to intensify the activity of metal centers needs a better knowledge of these oxides to change valence, by electron transfers in the outer or inner sphere.

The results presented in this study demonstrate that the polyoxometalates with mixed addenda (V, Mo, and W) can be modified by changing the ratio between metal ions. This allows obtaining new physical and chemical properties, such as electrical conductivity, magnetism, or catalytic activity.

Thus, this study contributes to the development of knowledge about this class of compounds and opens the possibility of exploring other Keggin polyoxometalates with different structures and functions. Given the vast array of transition metals and the numerous potential configurations of these compounds, this paves the way for further explorations.

This research demonstrates that polyoxometalates present inhibitor properties for S235 at 10^−2^ M or bigger concentration, which means that the additive limits, but does not cancel, the corrosion process, even if the efficiency is almost 90%.

The remarkable aspect is the effect of the studied polyoxometalates on SS304 stainless steel, where it proved to act as a passivant inhibitor at even 10^−5^ M concentration.

It can be concluded that in the case of austenitic stainless steels or other chromium and/or nickel alloys or other passivable metals/alloys, Keggin polyoxometalates (especially the ones with Mo) are remarkable oxidizing agents. They function as cathodic inhibitors, by forming protective films and bringing the metal or alloy into the passive zone, which is the safe zone, protected from corrosion.

In comparison to other inhibitors, such as chromates, polyoxometalates have very low toxicity; they accept electrons without significantly altering their structural integrity. They are desirable as passivators or protectors against corrosion because of these qualities for a large scale of steels, from mild to stainless steels.

## Figures and Tables

**Table 1:** Chemical composition of S235 and SS304 steel.

Steel	wt% C	wt% Si	wt% Mn	wt% Ni	wt% S	wt% P	wt% Cr	wt% N	wt% Cu
S235	0.22	0.05	0.06	0.3	0.04	0.04	0.3	0.012	0.3
SS304	0.07	0.75	2	8–10.5	0.03	0.045	17.5–19.5	0.1	-

**Table 2:** Electrochemical parameters of SS passivation curve in 0.5 M H_2_SO_4_ [29].

*E_corr_*, mV/SCE	*E_p_*, mV/SCE	*E_F_*, mV/SCE	*i_corr_*, μA/cm^2^	*i_p_*, μA/cm^2^
−440	−360	−250	1040	2.04

## Data Availability

Data are contained within the article.
